# *Wuchereria bancrofti*-infected individuals harbor distinct IL-10-producing regulatory B and T cell subsets which are affected by anti-filarial treatment

**DOI:** 10.1371/journal.pntd.0007436

**Published:** 2019-05-23

**Authors:** Manuel Ritter, Jubin Osei-Mensah, Linda Batsa Debrah, Alexander Kwarteng, Yusif Mubarik, Alexander Y. Debrah, Kenneth Pfarr, Achim Hoerauf, Laura E. Layland

**Affiliations:** 1 Institute of Medical Microbiology, Immunology and Parasitology, University Hospital Bonn, Bonn, Germany; 2 Kumasi Centre for Collaborative Research in Tropical Medicine (KCCR), Kumasi, Ghana; 3 Department of Clinical Microbiology, Kwame Nkrumah University of Science and Technology, Kumasi, Ghana; 4 Department of Biochemistry and Biotechnology, Kwame Nkrumah University of Science and Technology, Kumasi, Ghana; 5 Faculty of Allied Health Sciences, Kwame Nkrumah University of Science and Technology, Kumasi, Ghana; 6 German Centre for Infection Research (DZIF), partner site Bonn-Cologne, Bonn, Germany; Uniformed Services University of the Health Sciences, UNITED STATES

## Abstract

Despite worldwide mass drug administration, it is estimated that 68 million individuals are still infected with lymphatic filariasis with 19 million hydrocele and 17 million lymphedema reported cases. Despite the staggering number of pathology cases, the majority of LF-infected individuals do not develop clinical symptoms and present a tightly regulated immune system characterized by higher frequencies of regulatory T cells (Treg), suppressed proliferation and Th2 cytokine responses accompanied with increased secretion of IL-10, TGF-β and infection-specific IgG4. Nevertheless, the filarial-induced modulation of the host`s immune system and especially the role of regulatory immune cells like regulatory B (Breg) and Treg during an ongoing LF infection remains unknown. Thus, we analysed Breg and Treg frequencies in peripheral blood from Ghanaian uninfected endemic normals (EN), lymphedema (LE), asymptomatic patent (CFA+MF+) and latent (CFA+MF-) *W*. *bancrofti*-infected individuals as well as individuals who were previously infected with *W*. *bancrofti* (PI) but had cleared the infection due to the administration of ivermectin (IVM) and albendazole (ALB). In summary, we observed that IL-10-producing CD19^+^CD24^high^CD38d^high^ Breg were specifically increased in patently infected (CFA+MF+) individuals. In addition, CD19^+^CD24^high^CD5^+^CD1d^high^ and CD19^+^CD5^+^CD1d^high^IL-10^+^ Breg as well as CD4^+^CD127^-^FOXP3^+^ Treg frequencies were significantly increased in both *W*. *bancrofti*-infected cohorts (CFA+MF+ and CFA+MF-). Interestingly, the PI cohort presented frequency levels of all studied regulatory immune cell populations comparable with the EN group. In conclusion, the results from this study show that an ongoing *W*. *bancrofti* infection induces distinct Breg and Treg populations in peripheral blood from Ghanaian volunteers. Those regulatory immune cell populations might contribute to the regulated state of the host immune system and are probably important for the survival and fertility (microfilaria release) of the helminth.

## Author summary

Regulation of the host`s immune system by filarial nematodes is crucial for the fertility and survival of the nematode. Indeed, the majority of *W*. *bancrofti*-infected individuals are characterized by a regulated state including increased regulatory T cells (Treg), IL-10, TGF-β and filarial-specific IgG4 and suppressed Th2 cytokine responses. However, the functional role of Treg populations and regulatory B cells (Breg) during filarial infection remains unknown. Thus, in this study we investigated whether *W*. *bancrofti*-infected individuals from Ghana harbored distinct Breg and Treg populations which might be important for filarial-specific immunomodulation. Overall, this study shows that *W*. *bancrofti* induces distinct Breg populations, especially in patently (microfilaremic) infected individuals who presented significantly increased frequencies of IL-10-producing CD19^+^CD24^high^CD38d^high^ Breg. Furthermore, clearance of the infection, due to anti-filarial treatment, returned these regulatory immune cells to homeostatic levels showing that an ongoing filarial infection is important for the activation of distinct Breg and Treg subsets. Those regulatory immune cell subsets are a part of a complex system which are induced by filarial nematodes to modulate the host`s immune system and maintain long-term survival.

## Introduction

Helminths like filarial nematodes are tropical parasitic worms and the infections that they induce are classified as neglected tropical diseases (NTDs). Filarial infections are vector-borne diseases which are transmitted by blood-feeding insects that are common in tropical and subtropical countries. Although the majority of filarial infections remain in a regulated state, long-term chronic infections can cause overt diseases and individuals suffering from filarial-induced diseases are stigmatized and endure immense social and psychological burdens as well as financial losses which contribute to poverty [1]. For example, lymphatic filariasis (LF) is caused by *Wuchereria bancrofti* and *Brugia* spp. and can lead to the development of hydrocele, lymphedema, lymphangitis and elephantiasis causing a major public health problem and an overall elevation in disability-adjusted life years (DALY). Before mass drug administration (MDA) commenced, approximately 120 million people were infected with LF, and 40 million people suffered from disease-related pathologies. Therefore, the World Health Organization launched the Global Programme to Eliminate LF (GPELF) and MDA measures have cured or prevented 96 million new cases of LF over the last 13 years. It is now estimated that 68 million people are still infected and there are 19 million hydrocele and 17 million lymphedema cases [2].

As mentioned above, whereas a portion of humans develop severe forms of disease-related symptoms the majority of individuals retain a homeostatic and regulated state which is essential for the long-term survival of filariae [3–5]. Regulatory immune cells play a crucial role in the regulation of immune responses and indeed higher frequencies of regulatory T cells (Treg) were observed in LF-infected microfilaremic (MF+) and microfilariae negative (MF-) individuals compared to uninfected adolescents and individuals with lymphedema [6, 7]. In addition, *in vitro* stimulation assays revealed that Tregs obtained from MF+ individuals suppressed proliferation and Th2 cytokine responses [8]. Furthermore, it was shown that the modified Th2 responses in MF+ individuals are accompanied with higher frequencies of Treg and alternatively activated macrophages as well as increased secretion of IL-10, TGF-β and infection-specific IgG4: all promoting parasite survival [9, 10]. In addition to Treg, regulatory B cells (Breg) have been widely recognized as negative regulators of immune responses controlling autoimmunity and inflammation in suppressing pathological immune responses primarily through the secretion of IL-10 [11]. Indeed, it was shown that helminth infections induce IL-10-producing Breg populations [12–14] but the role of such immune cell subsets during filarial infection remains unclear.

Thus, to decipher the role of regulatory immune cell subsets during LF, we analysed Breg and Treg frequencies in peripheral blood from uninfected endemic normals (EN), asymptomatic patent (CFA+MF+) and latent (CFA+MF-) *W*. *bancrofti*-infected individuals in Ghana. In addition, to elucidate the prevalence of distinct Breg and Treg subsets in individuals who had cleared the infection but suffer from *W*. *bancrofti*-induced clinical symptoms, we also profiled individuals with lymphedema (LE) who were CFA-MF-. Since MDA treatment against LF has been applied in Ghana since 2000 [15], we also analysed peripheral blood from individuals which were previously infected with *W*. *bancrofti* (PI) but had cleared the infection due to the administration of ivermectin (IVM) and albendazole (ALB). The composition and inclusion of the different patient groups allowed a detailed analysis of regulatory immune cell subsets in *W*. *bancrofti*-affected individuals (CFA+MF+, CFA+MF-, LE, PI) in comparison to EN. We observed that all *W*. *bancrofti*-infected individuals had significantly increased CD19^+^CD24^high^CD5^+^CD1d^high^ and CD19^+^CD5^+^CD1d^high^IL-10^+^ Breg as well as CD4^+^CD127^-^FOXP3^+^ Treg frequencies in the peripheral blood whereas IL-10-producing CD19^+^CD24^high^CD38d^high^ Bregs were exclusively increased in patently infected (CFA+MF+) individuals. In addition, anti-filarial treatment and clearance of infection (PI group) lead to the reduction of Breg and Treg subsets to levels comparable with those from EN. In summary, the results obtained from this study show that distinct Breg and Treg subsets are induced during an ongoing *W*. *bancrofti* infection but return to homeostatic levels upon clearance of infection indicating a potential contribution to the filarial-specific immunity and survival of the parasite.

## Methods

### Ethics statement

The studies were approved by the Committee on Human Research, Publications and Ethics at the School of Medical Sciences of the Kwame Nkrumah University of Science and Technology (KNUST), and Komfo Anokye Teaching Hospital, Kumasi, Ghana (CHRPE/AP/022/16), as well as by the Ethics Committee of the University Hospital of Bonn, Germany (018/12). Permission was also obtained from the Nzema East and Ahanta West District Health Directorates, Ghana. Before recruitment and sample collection commenced, meetings were held in the communities to explain in detail the purpose and procedures of the study. Verbal consent to perform the study in the villages was obtained from community leaders, i.e., chiefs and elders of the selected communities, and written informed consent was obtained from all participants. The study was undertaken according to the principles of the Helsinki Declaration of 1975 (as revised 2008).

### Study population

In 2009, a case control study was conducted in 1774 Ghanaian volunteers within the health districts Nzema East and Ahanta West of the Western region of Ghana to identify genetic biomarkers which are associated with different manifestations of lymphatic filariasis (LF). In 2015, a total of 223 individuals from the initial study agreed to a follow up study and provided peripheral whole blood to characterize regulatory immune cell populations using flow cytometry technique.

### Parasitic and lymphedema assessment

To assess *W*. *bancrofti* infection, night blood was obtained from the participants to determine the presence of MF since the nematode has a nocturnal periodic activity. Finger prick blood test, thick blood film smears and Sedgewick rafter counting technique were all performed. For the thick blood film technique, peripheral whole blood was applied on a glass slide, stained with Giemsa and examined for MF under the microscope at x10 magnification. In addition, 100μl whole blood was mixed with 900μl of 3% acetic acid, poured onto a Sedgewick rafter counting chamber (VWR, Langenfeld, Germany) and MF counts were examined using a microscope at x10 magnification. Furthermore, circulating filarial antigen (CFA) was detected using immunochromatographic card test (ICT) from the BinaxNOW^®^ Filariasis kit (Alere, Cologne, Germany) according to the manufactures description. Lymphedema (LE) individuals were characterized based on the presence of oedema on the upper and lower limb extremities according to the “Basic Lymphedema Management Guidelines” established by Dreyer and colleagues [16]. At the time of sampling, LE individuals tested negative for both CFA and MF parameters, confirming previous studies showing that individuals suffering from lymphedema are usually MF and antigen negative [6, 17]. In addition, since no red clay soils derived from volcanic deposits are present in the study region, podoconiosis-induced lymphedema cases were not observed [18, 19]. A Malaria Pf Ag rapid test (Guangzhou Wondfo Biotech Co. Ltd, Guangzhou, China) was further applied according to the manufacturer’s instructions to determine *Plasmodium* infection. Other filarial infections were ruled out via blood smear analysis (e.g. *Mansonella perstans*) or absence at the study site (*Onchocerca volvulus*).

### *In vitro* stimulations of peripheral whole blood cells

100μl whole blood from the participants were plated onto 96-well culture plates (Greiner Bio-One GmbH, Frickenhausen, Germany) and cultivated in 100μl RPMI-1640 medium (Sigma-Aldrich, Munich, Germany) including 10% bovine calf serum (BCS, Sigma-Aldrich). Whole blood cultures were then left un-stimulated or re-stimulated with eBioscience^™^ cell stimulation cocktail (PMA; Thermo Fisher Scientific, Schwerte, Germany) for 4h at room temperature. Thereafter, regulatory immune cell composition and function was analysed using flow cytometry.

### Analysis of regulatory immune cell composition and function in peripheral whole blood

To obtain whole blood cells from the in vitro cultures, plates were centrifuged and supernatants removed. Red blood cells were then eliminated from the cultures using a red blood cell lysis buffer (Biolegend, San Diego, USA) and remaining cells were fixed and permeabilized using eBioscience^™^ fixation/permeabilization concentrate and permeabilization buffer (Thermo Fisher Scientific) according to the manufacture`s description. Thereafter, cells were stained with combinations of fluorophore (FITC, PE, PE-Cy7, APC)-conjugated anti-human CD1d (clone 51.1), CD4 (clone RPA-T4), CD5 (clone UCHT2), CD19 (clone HIB19), CD24 (clone eBioSN3 (SN3 A5-2H10)), CD38 (clone HIT2), CD127 (clone eBioRDR5), FOXP3 (clone 236A/E7), HELIOS (clone 22F6), IL-10 (clone JES3-9D7), eBioscience^™^ monoclonal antibodies from Thermo Fisher Scientific and CD304 (Neuropilin-1, clone 12C2) monoclonal antibody from Biolegend. Stained samples were stored at 4°C and kept in the dark. Within 7 days, the samples were transported to the Kumasi Centre for Collaborative Research in Tropical Medicine (KCCR) in Kumasi, Ghana and acquired using the BD Accuri^™^ Flow cytometer (BD Bioscience). Afterwards antibody expression levels were analysed using the FlowJo v10 software (FlowJo, LLC, USA). An overview of the analysed regulatory immune cell subsets with their corresponding flow cytometry markers is shown in Table 1.

**Table 1:** Analysed regulatory immune cell subsets and their corresponding flow cytometry markers.

Regulatory immune cell subset	Flow cytometry marker	Appearance
Regulatory B cell	CD19^+^CD24^high^CD5^+^CD1d^high^	Fig 1, S1 Fig
IL-10 producing regulatory B cells (B10)	CD19^+^CD5^+^CD1d^high^IL-10^+^	Fig 2, S3 Fig
IL-10-producing immature B cells	CD19^+^C24^high^CD38^high^IL-10^+^	Fig 3, S4 Fig
Thymic-derived and peripherally induced regulatory T cells (tTreg and pTreg)	CD4^+^CD127^-^FOXP3^+^ Neuropilin-1/Helios^+^	Fig 4, S5 Fig

### Statistical analysis

Statistical analyses were performed using the software SPSS (IBM SPSS Statistics 22; Armonk, NY) and the PRISM 5 programme (GraphPad Software, Inc., La Jolla, USA). Variables did not meet assumptions to allow parametric analysis, therefore to compare more than two groups a Kruskal-Wallis-test was performed and, if significant, followed by a Dunn`s multiple comparison test for a further comparison of the groups. The Spearman`s rank correlation coefficient was applied to analyse rank correlations between two variables. Finally, stepwise multiple logistic regression analysis was performed to decipher possible confounders like gender, age or rounds of MDA on the immunological results. P-values of 0.05 or less were considered significant.

## Results

### Study population

An initial case control study performed in 2009 in the health districts Nzema East revealed 318 patent (CFA+MF+) and 397 latent (CFA+MF-) *W*. *bancrofti* infections as well as 246 lymphedema (LE) and 349 endemic normals (EN). MDA programmes (400mg ALB + 200μg/kg IVM once a year) in these areas by the Ministry of Health were running from 2009. Based on the initial study we re-visited the health districts in 2015 to determine *W*. *bancrofti* infections upon implementation of anti-filarial treatment and to analyse the composition and function of regulatory B and T cell subsets using flow cytometry. In total, we obtained 54 EN, 41 CFA+MF-, 13 CFA+MF+, 50 LE and 65 individuals who were previously infected (PI) with *W*. *bancrofti* (CFA+MF- or CFA+MF+) in 2009 but were now classified as CFA-MF-. All participants were negative for *Plasmodium* or other filarial infections. An overview about the characteristics of the study population is depicted in Table 2 and S1 Table.

**Table 2:** Characteristics of study population. According to their diagnostic status, individuals were categorized as endemic normal (EN), latent (CFA+MF-) or patent (CFA+MF+) *W*. *bancrofti*-infected and lymphedema (LE) as well as previously infected individuals (PI) who had cleared the infection. Table 2 shows total sample size, gender, health district and community as well as age, microfilariae (MF) numbers and MDA treatment rounds since 2009 which are given as mean, median and range.

	EN	CFA+MF-	CFA+MF+	LE	PI
Sample size (n)	54	41	13	50	65
Mean age [years]	45.7	43.6	43.2	50.2	41.4
Median age [years]	45.5	42	45	50	38
Range age [years]	22–80	28–64	24–58	25–70	23–78
Gender [Female:Male]	46:8	21:20	1:12	42:8	37:28
Health district	Ahanta West	Ahanta West, Nzema East	Ahanta West, Nzema East	Ahanta West, Nzema East	Ahanta West
Community	Achowa, Apatano, Asamasa, Asemkow, Busua, Butre, Cape 3 Point, Kantakrom	Adukrom, Agyan, Akatakyi, Akonu, Apatano, Asemkow, Bakanta, Butre, Cape 3 Point, Dixcove, Domunli, Sanwoma	Agyan, Akatakyi, Akonu, Apatano, Asanta, Cape 3 Point, Domunli, Samwona	Achowa, Akatakyi, Apatano, Asamasa, Asemkow, Butre, Busua, Cape 3 Point, Kantakrom, Miamia	Achowa, Akyinim, Apatano, Asemkow, Busua, Butre, Kantakrom
Mean microfilaria count [MF/ml]	0	0	243.6	0	0
Median microfilaria count [MF/ml]	0	0	123.5	0	0
Range microfilaria count [MF/ml]	0	0	1–984	0	0
Mean MDA rounds	6	3.8	1.8	6.4	5.2
Median MDA rounds	5	3	0	6	4
Range MDA rounds	0–10	0–10	0–6	2–10	1–10

### Increased frequencies of regulatory B cells in *W*. *bancrofti*-infected and lymphedema individuals

**Fig 1:**
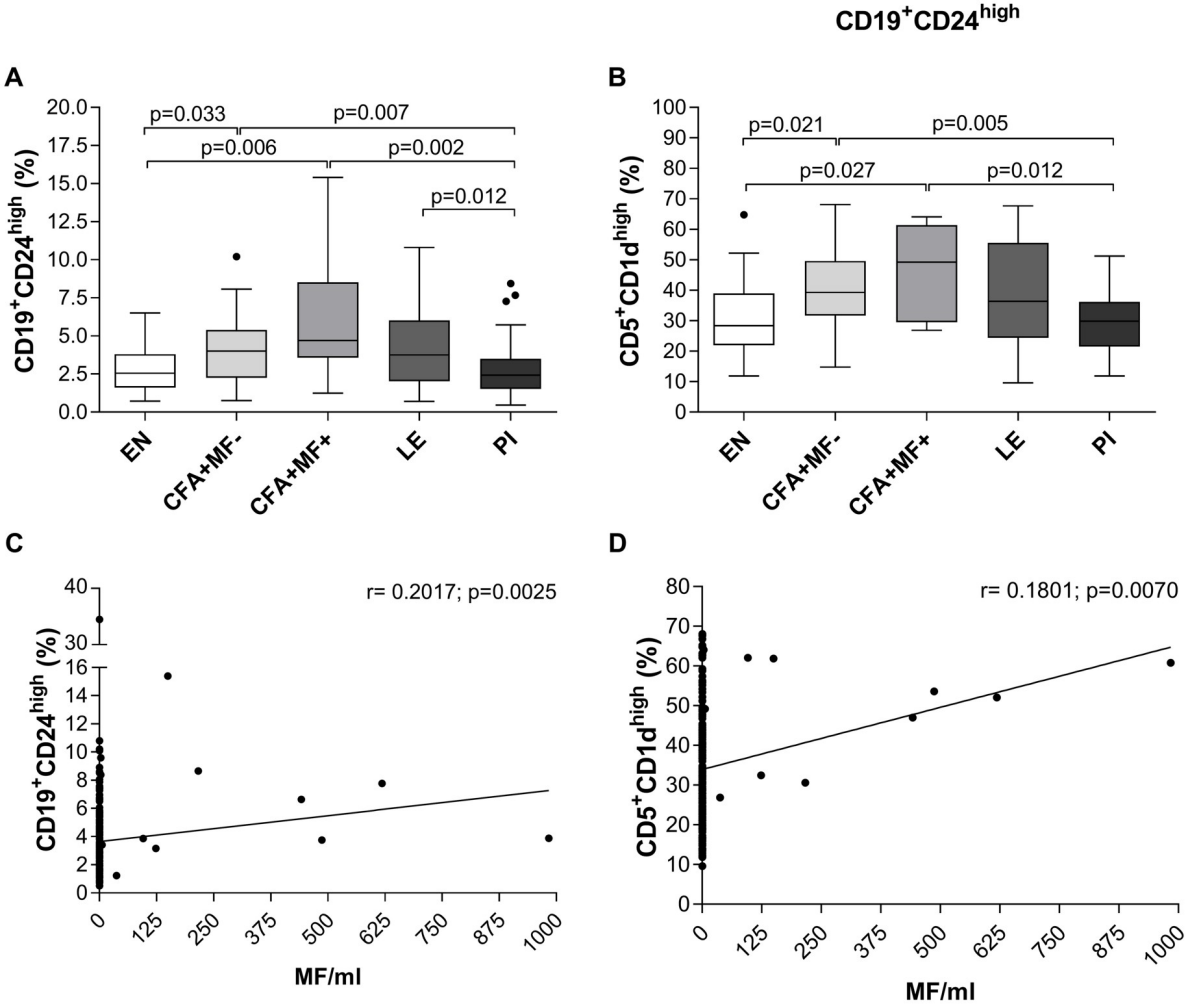
Increased CD19^+^CD24^high^CD5^+^CD1d^high^ Breg frequencies in peripheral blood of *W*. *bancrofti*-infected and LE individuals. Using flow cytometry, peripheral whole blood cells from endemic normals (EN; n = 54), latent (CFA+MF-; n = 41) and patent (CFA+MF+; n = 13) *Wuchereria bancrofti*-infected, lymphedema (LE, n = 50) and previously infected individuals (PI; n = 65) were analyzed for frequencies (%) of (**A**) CD19^+^CD24^high^ regulatory B cells expressing (**B**) CD5^+^CD1d^high^. Graphs show box whiskers with median, interquartile ranges and outliers. Statistical significances between the indicated groups were obtained after a Kruskal-Wallis-test followed by a Dunn`s multiple comparison post hoc analysis. In addition, Spearman correlations were performed between MF counts and (**C**) CD19^+^CD24^high^ or (**D**) CD19^+^CD24^high^CD5^+^CD1d^high^ frequencies.

Peripheral whole blood was obtained from the participants (Table 2) and frequencies of regulatory B (Breg) and T cell (Treg) populations were analysed using flow cytometry according to the applied gating strategy (S1 and S3–S5 Figs). The frequencies of CD19^+^CD24^high^ (Fig 1A) and CD19^+^CD24^high^CD5^+^CD1d^high^ (Fig 1B) Breg subsets were significantly increased in latent (CFA+MF-) and patent (CFA+MF+) *W*. *bancrofti*-infected individuals when compared to EN and PI. Interestingly, a heamatological study in India reported that whole blood cell counts were increased in individuals presenting filariasis [20]. However, flow cytometry-based analysis of lymphocytes, according to the applied gating strategy here (S1 Fig), showed equal lymphocyte frequencies between the different groups (S2 Fig). Therefore, Breg subsets were induced by *W*. *bancrofti* infection and not the result of an overall lymphocyte expansion. Moreover, microfilaremic (CFA+MF+) individuals presented an overall higher frequency of those subsets and further analysis revealed positive correlations between MF counts and measured CD19^+^CD24^high^ (r = 0.2017, p = 0.0025) and CD19^+^CD24^high^CD5^+^CD1d^high^ (r = 0.1801, p = 0.0070) Breg subsets (Fig 1C and 1D, respectively). These findings show that *W*. *bancrofti* infections, especially patent ones, induce distinct Breg subsets and interestingly, in the PI cohort these population had levels comparable to the EN group indicating that they had returned to homeostatic levels.

### Elevated systemic levels of IL-10-producing regulatory B cells in *W*. *bancrofti*-infected individuals

**Fig 2:**
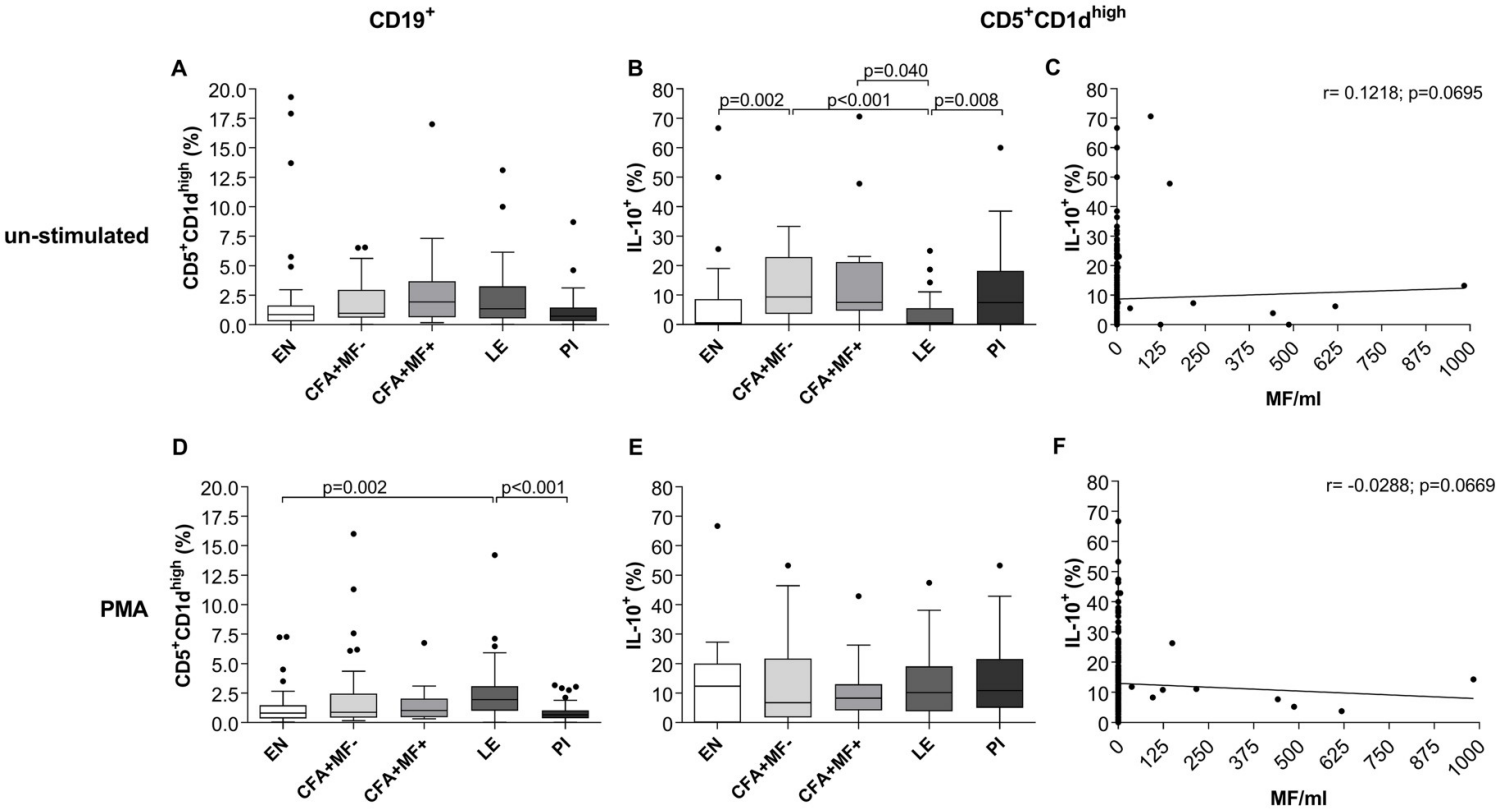
Increased CD19^+^CD5^+^CD1d^high^IL-10^+^ Breg frequencies in peripheral blood of *W*. *bancrofti*-infected individuals. Freshly isolated peripheral whole blood cells (100μl/well) from endemic normals (EN; n = 54), latent (CFA+MF-; n = 41) and patent (CFA+MF+; n = 13) *Wuchereria bancrofti*-infected, lymphedema (LE, n = 50) and previously infected individuals (PI; n = 65) were cultivated in 10% FCS/RPMI-1640 medium (100μl/well) and left either (**A-C**) un-stimulated or (**D-F**) cultured with eBioscience^™^ cell stimulation cocktail (PMA) for 4 hours at room temperature. Thereafter, peripheral blood cells were analyzed for frequencies (%) of CD19^+^ B cells expressing (**A, D**) CD5^+^CD1d^high^ and CD19^+^CD5^+^CD1d^high^ regulatory B cells expressing (**B, E**) IL-10. Graphs show box whiskers with median, interquartile ranges and outliers. Statistical significances between the indicated groups were obtained after a Kruskal-Wallis-test followed by a Dunn`s multiple comparison post hoc analysis. In addition, Spearman correlations were performed between MF counts and frequencies of CD19^+^CD24^high^CD38^high^IL-10^+^ which were either (**C**) un-stimulated or (**F**) PMA stimulated.

Since *W*. *bancrofti* infection induces Breg accumulation in the periphery, we further deciphered the functional role of Breg subsets. Bregs are predominately identified on their ability to produce IL-10 [11] which regulates autoimmunity [21] and suppresses T cell and cytokine responses [22, 23]. In mice, IL-10-producing CD19^+^CD5^+^CD1d^high^ Bregs are called B10 cells which were shown to be induced by LPS or PMA stimulation [24]. Therefore, peripheral whole blood cells were either left untreated (Fig 2A–2C) or stimulated with PMA (Fig 2D–2F) and the frequency of IL-10-producing CD19^+^CD5^+^CD1d^high^ Bregs were analysed according to the applied gating strategy (S3 Fig). Without *ex vivo* stimulation the frequencies of CD19^+^CD5^+^CD1d^high^ Bregs were by tendency increased in *W*. *bancrofti*-infected and LE individuals (Fig 2A) when compared to EN and PI groups. IL-10-producing CD19^+^CD5^+^CD1d^high^ Bregs however, were significantly increased in *W*. *bancrofti*-infected groups compared to EN or LE (Fig 2B). In contrast to the significantly decreased frequency of CD19^+^CD24^high^CD5^+^CD1d^high^ cells in PI individuals (Fig 1B), IL-10-producing CD19^+^CD5^+^CD1d^high^ frequencies within un-stimulated peripheral whole blood cells from PI individuals were comparable to *W*. *bancrofti*-infected individuals. In addition, in LE individuals, frequencies of IL-10-producing CD19^+^CD5^+^CD1d^high^ Bregs were significantly reduced when compared to *W*. *bancrofti-*infected and PI individuals (Fig 2B), showing that CD19^+^CD5^+^CD1d^high^ Bregs were functionally impaired. Further analysis revealed no significant correlation between MF counts and un-stimulated CD19^+^CD5^+^CD1d^high^IL-10^+^ frequencies (r = 0.1218, p = 0.0669; Fig 2C). However, PMA *ex vivo* stimulation again revealed significantly increased frequencies of CD19^+^CD5^+^CD1d^high^ Bregs in LE compared to EN and PI individuals (Fig 2D) but no differences could be observed between the different cohorts with regards to IL-10 production (Fig 2E). Again, no significant correlation between MF counts and PMA-stimulated CD19^+^CD5^+^CD1d^high^IL-10^+^ frequencies were observed (r = -0.0288, p = 0.0669; Fig 2F).

### IL-10-producing immature B cells are induced in patent *W*. *bancrofti*-infected individuals

**Fig 3:**
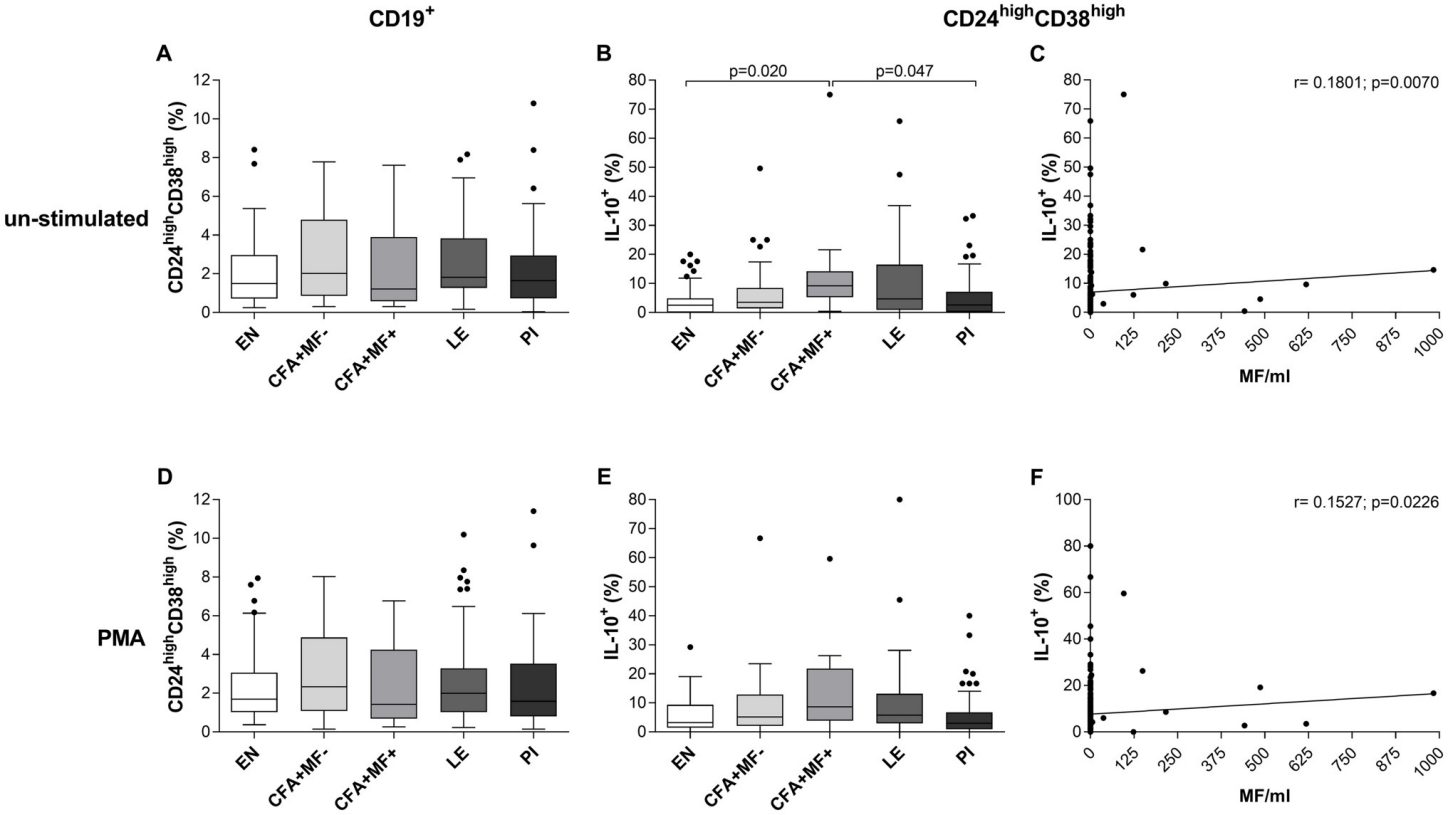
Increased CD19^+^CD24^high^CD38d^high^IL-10^+^ Breg frequencies in peripheral blood of patent *W*. *bancrofti*-infected individuals. Freshly isolated peripheral whole blood cells (100μl/well) from endemic normals (EN; n = 54), latent (CFA+MF-; n = 41) and patent (CFA+MF+; n = 13) *Wuchereria bancrofti*-infected, lymphedema (LE, n = 50) and previously infected individuals (PI; n = 65) were cultivated in 10% FCS/RPMI-1640 medium (100μl/well) and left either (**A-C**) un-stimulated or (**D-F**) cultured with eBioscience^™^ cell stimulation cocktail (PMA) for 4 hours at room temperature. Thereafter, peripheral blood cells were analyzed for frequencies (%) of CD19^+^ B cells expressing (**A, D**) CD24^high^CD38^high^ and CD19^+^CD24^high^CD38^high^ immature B cells expressing (**B, E**) IL-10. Graphs show box whiskers with median, interquartile ranges and outliers. Statistical significances between the indicated groups were obtained after a Kruskal-Wallis-test followed by a Dunn`s multiple comparison post hoc analysis. In addition, Spearman correlations were performed between MF counts and frequencies of CD19^+^CD24^high^CD38^high^IL-10^+^ which were either (**C**) un-stimulated or (**F**) PMA stimulated.

Besides CD19^+^CD5^+^CD1d^high^IL-10^+^ Breg populations, IL-10-producing immature B cells (CD19^+^CD24^high^CD38^high^IL-10^+^) are a crucial immunomodulating cell subset in humans since they suppress effector T cell responses as well as Th1 and Th17 differentiation, promote the conversion of CD4^+^ T cells into regulatory T cells (Treg) and type 1 regulatory T cells (Tr1) and play additional roles during autoimmunity, HIV infection and graft-versus-host disease [21, 25–27]. To analyse IL-10-producing immature B cell frequencies in *W*. *bancrofti*-infected individuals, peripheral whole blood cells were left either untreated (Fig 3A–3C) or stimulated with PMA (Fig 3D–3F) and frequencies were analysed according to the applied gating strategy (S4 Fig). Whereas no differences in the frequency of CD19^+^CD24^high^CD38^high^ Breg subsets could be observed between the groups (Fig 3A), CFA+MF+ had significantly increased CD19^+^CD24^high^CD38^high^IL-10^+^ frequencies when compared to EN and PI without *ex vivo* stimulation (Fig 3B). In addition, albeit weak, further analysis revealed a positive correlation between MF counts and un-stimulated CD19^+^CD24^high^CD38^high^IL-10^+^ frequencies (r = 0.1801, p = 0.0070; Fig 3C). Again, upon PMA *ex vivo* stimulation, no differences of the frequencies could be observed between the different groups (Fig 3D and 3E), although positive correlation between MF counts and PMA-stimulated CD19^+^CD24^high^CD38^high^IL-10^+^ frequencies were significant (r = 0.1527, p = 0.0226; Fig 3F). Overall, these findings suggest that besides CD19^+^CD5^+^CD1d^high^IL-10^+^ Bregs, IL-10-producing immature B cells are also induced by *W*. *bancrofti* infection and that individuals from the different cohorts have the same potential to produce IL-10 upon *ex vivo* stimulation.

### *W*. *bancrofti* induces peripheral induced Tregs

**Fig 4:**
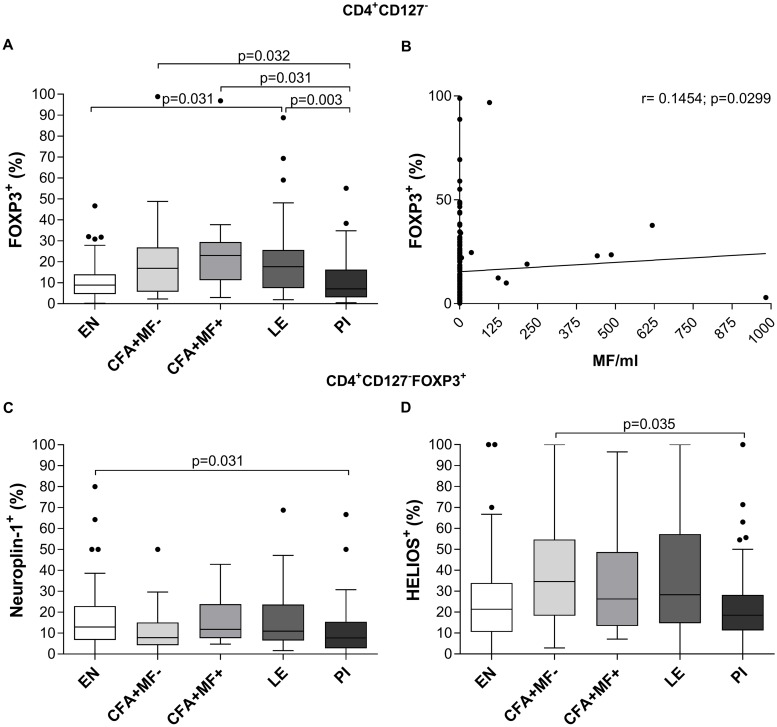
Increased CD4^+^CD127^-^FOXP3^+^ Treg frequencies in peripheral blood of *W*. *bancrofti*-infected and LE individuals. Using flow cytometry, peripheral whole blood cells from endemic normals (EN; n = 54), latent (CFA+MF-; n = 41) and patent (CFA+MF+; n = 13) *Wuchereria bancrofti*-infected, lymphedema (LE, n = 50) and previously infected individuals (PI; n = 65) were analyzed for frequencies (%) of (**A**) CD4^+^CD127^-^FOXP3^+^ regulatory T cells expressing (**C**) Neuropiln-1 and (**D**) HELIOS. Graphs show box whiskers with median, interquartile ranges and outliers. Statistical significances between the indicated groups were obtained after a Kruskal-Wallis-test followed by a Dunn`s multiple comparison post hoc analysis. (**B**) Spearman correlation was performed between MF counts and CD4^+^CD127^-^FOXP3^+^ regulatory T cell frequencies.

Besides Bregs, Treg (CD4^+^CD25^+^ and/or CD4^+^FOXP3^+^) were shown to be induced during human filarial infections [8, 28, 29], but the role of distinct Treg subsets and their precise role during lymphatic filariasis remains unclear. FOXP3^+^ Tregs can be divided into natural or thymic-derived (tTreg) and peripherally induced Treg (pTreg) [30]. Furthermore, Neuropilin-1 and HELIOS were declared as potential markers for tTreg [31, 32] and were used to discriminate FOXP3^+^ Treg populations here, see applied gating strategy (S5 Fig). Indeed, without *ex vivo* stimulation, CD4^+^CD127^-^FOXP3^+^ Treg frequencies were increased in the entire *W*. *bancrofti*-infected cohort and LE individuals when compared to EN and PI (Fig 4A), confirming that filarial infections promote Treg accumulation [8, 28, 29]. In addition, further analysis revealed a positive correlation between MF numbers and CD4^+^CD127^-^FOXP3^+^ frequencies (r = 0.1454, p = 0.0299; Fig 4B). In regards to the discrimination of tTreg and pTreg, Neuropilin-1 and HELIOS expression on CD4^+^CD127^-^FOXP3^+^ Treg remained unaltered between CFA+MF+, CFA+MF- and LE, but the PI group showed significantly decreased Neuropilin-1 and HELIOS frequencies compared to the EN and CFA+MF- group (Fig 4C and 4D). Since Neuropilin-1 expression was unaltered in the *W*. *bancrofti*-infected and LE individuals it suggests that the increased frequency of CD4^+^CD127^-^FOXP3^+^ cells (Fig 4A) are due to the induction of pTreg.

The overall data set of the study is shown in S1 Table.

## Discussion

The underlying mechanisms as to why many LF-infected individuals remain in a homeostatic state are still not fully resolved. No doubt multiple subtle triggers and interactions by this nematode on the host contribute to this unique relationship. Although, B cell immunoregulatory mechanisms have been reported in a murine model of *Brugia pahangi* [31], this study documents primary evidence of functional Breg populations in *W*. *bancrofti* infections and reveals how distinct Breg subsets contribute to this overall picture in man: active functional Breg populations that subside upon treatment. In terms of immune-regulation several studies have shown that besides increased levels of IL-10, TGF-β, filarial-specific IgG4 and frequencies of alternatively activated macrophages [9, 10], filarial-infected individuals harbour increased Treg frequencies too [7] with higher expression levels of FOXP3, CTLA-4, TGF-β and PD-1 on isolated PBMCs [10]. Expanding on those findings, we show here that CD4^+^CD127^-^FOXP3^+^ Treg frequencies were higher in *W*. *bancrofti*-infected individuals and this included the LE group. Moreover, individuals who had cleared the infection due to MDA participation had Treg frequencies comparable to EN indicating that the cells are only required during active infections. In addition, there are two subpopulations of FOXP3^+^ Treg called tTreg and pTreg [30], but their role in *W*. *bancrofti* infections remains uncertain [28]. Therefore, we deciphered the frequencies of these subsets using the markers Neuropilin-1 and HELIOS [32, 33] but revealed no differences in Neuropilin-1 and HELIOS expression on CD4^+^CD127^-^FOXP3^+^ Tregs. Interestingly, more recent studies have indicated that HELIOS is not a definite marker for tTreg [34, 35] and thus, these findings need to be critically assessed. Nevertheless, since the frequencies of Neuropilin-1 were equal between the CFA+MF+ and CFA+MF- we suggest that the CD4^+^CD127^-^FOXP3^+^ Treg levels in peripheral blood of *W*. *bancrofti*-infected individuals depend on the induction of pTreg which indeed were shown to be generated in the periphery upon antigen exposure [30]. And moreover were classified as effective suppressors [36]. In association, CD4^+^CD25^high^ Tregs obtained from microfilaremic (MF+) *Brugia malayi*-infected individuals suppressed proliferation and Th2 cytokine responses [8] and a limitation to the current work is the lack of functional suppression assays using the observed regulatory populations. Therefore, future studies should focus on identifying filarial-specific inhibition by those subsets using *W*. *bancrofti* or *Brugia* antigen extracts. Besides the CD4^+^CD25^+^FOXP3^+^CD127^-^ Treg population IL-10-producing regulatory type 1 T cells (Tr1) were detected in filarial-infected individuals [7, 37] whereas we recently showed that CD4^+^α/βTCR^+^CD49b^+^LAG3^+^ Tr1 were decreased in peripheral blood of *M*. *perstans*-infected individuals [29]. However, further in depth analysis including Treg markers like CD103, CTLA-4, GITR, ICOS, LAG-3 and TGF-β which were shown to be important for the characterization and function of Treg [30, 38–40] need to be performed to decipher the role of pTreg during filarial infection in more detail. Indeed, one limitation of this study was that flow cytometry analysis of peripheral blood was performed in Ghana using the BD Accuri^™^ Flow cytometer which only allowed 4 colour-based analysis and thus, as mentioned above, characterization of the different regulatory immune cell populations was restricted. Nevertheless, this study indicate that *W*. *bancrofti* infection induces pTreg, which were shown to mediate their suppressive function through CTLA-4, GITR, LAG-3, and membrane-bound TGF-β [39–42].

Besides Tregs, the expanding family of Bregs and their role in suppressing pathological immune responses and during helminth infections has recently been recognized [11, 14, 43, 44]. For example, it was shown that various helminth infections induce IL-10-producing Breg populations [12–14, 45] which were shown to be antigen-specific during chronic schistosomiasis [46, 47], but the distinct role of Breg populations during *W*. *bancrofti* infection has remained largely unclear. Recently, we showed that *M*. *perstans*-infected individuals harbour high frequencies of CD19^+^CD24^high^CD38^high^CD1d^high^ Bregs when compared to uninfected individuals [29] demonstrating that Breg populations are part of the cellular composition that retains a balanced immune reaction to *M*. *perstans* infections. Furthermore, we now reveal that *W*. *bancrofti*-infected individuals had increased CD19^+^CD24^high^CD5^+^CD1d^high^, IL-10-producing CD19^+^CD5^+^CD1d^high^ and CD19^+^CD24^high^CD38d^high^ Breg frequencies in peripheral blood. Interestingly, it was shown that patients with multiple sclerosis (MS), who were co-infected with helminths had increased frequencies of IL-10-producing CD19^+^CD1d^high^ B cells which suppressed T cell proliferation and IFN-γ production leading to a better clinical outcome in regards to MS [13]. With regards to the observed IL-10-producing CD19^+^CD24^high^CD38d^high^ Breg population, previous studies have shown that immature B cell populations (CD19^+^CD24^high^CD38d^high^) can produce high amounts of IL-10 upon CD40 engagement leading to suppression of Th1 and Th17 differentiation and conversion of CD4^+^ T cells into Treg and Tr1 cells [21, 25]. Consequently, this IL-10 producing immature B cell population was shown to influence and modulate immune responses during autoimmunity, HIV infection and graft-versus-host disease [11, 21, 25–27]. In detail, this Breg subset was shown to maintain tolerance and long term remission during autoimmunity [48, 49] and was associated with reduced rejection rates upon kidney transplantation [50] but also may contribute to immune dysfunction in HIV infection through the suppression of HIV-1 specific CD8^+^ T cell responses [26]. However, here we show that this IL-10-producing immature B cell population is present in peripheral blood of *W*. *bancrofti*-infected individuals and was positively correlated with MF release suggesting that the induction of this Breg population promotes fertility and survival of the parasite.

With regards to the observed CD19^+^CD24^high^CD5^+^CD1d^high^ and IL-10-producing CD19^+^CD5^+^CD1d^high^ Breg populations, to our knowledge this is the first study that shows the presence of so-called B10 cells in *W*. *bancrofti*-infected individuals. Several studies observed that B10 cells can be induced upon LPS or PMA stimulation in mice and have been shown to suppress inflammation [24]. As with other Breg subsets, the suppressive function of B10 cells depend on CD40 engagement [51] and several experimental mouse models have proven the efficacy of B10 cells in dampening autoimmunity [52, 53]. However, precursor B10 (B10_Pro_) and B10 cells were also identified in humans and it is suggested that their development depend on LPS and CpG stimulation and CD40 ligation [23, 54]. Since we observed increased B10 cell frequencies in *W*. *bancrofti*-infected individuals without *ex vivo* stimulation we suggest that especially the MF provide the stimuli that drive B10 development in peripheral blood. Indeed, several studies already showed that inflammatory responses by filariae are mediated by TLR-inducing activity from the endosymbiotic *Wolbachia* bacteria [55–57] which are released from dying MF [58–60]. This study shows that there is an elevation of distinct IL-10-producing Breg subsets during *W*. *bancrofti* infection. These Breg subsets could potentially regulate host immunity through the secretion of IL-10 which has been shown to induce immunosuppressive alternatively activated macrophages as well as IgG4, which inhibits the function of various immune cells [9, 61–64]. In addition, *Brugia pahangi* infection experiments in mice revealed that B cell populations and IL-10 secretion play an important role in filarial-driven immunomodulation [31]. Nevertheless, further studies need to decipher whether these distinct Bregs mediate their suppressive function through other molecules like TGF-β that were shown to modulate immune responses during patent filarial infections [28, 42]. Although the analysed regulatory immune cell subsets comprised only a small percent of the overall lymphocyte population, we do consider that they are relevant in shaping host immunity during *W*. *bancrofti* infection since levels returned to those found in EN after infections were cleared. Indeed, recent studies showed that serial single cell adoptive transfer experiments and even low numbers of CD8^+^ T cells were effective against *Listeria monocytogenes* in a murine infection model [65, 66], indicating that specificity, education and functional relevance is more critical than cell number.

Ghana was one of the first countries in which the MDA against LF was implemented and programmes now cover the whole country [15]. Thus, we were able to analyse whether MDA affects the frequencies of regulatory immune cells in peripheral blood by revisiting our study cohort from 2009. During this unique opportunity we recruited 65 individuals who were previously infected (PI) with *W*. *bancrofti* (CFA+MF- or CFA+MF+) but were now classified as CFA-MF-. Indeed, treatment and thus clearance of infection lead to the reduction of Breg and Treg populations in the peripheral blood showing that ongoing *W*. *bancrofti* infections and especially MF release, appears to maintain regulatory immune cell development. In addition, sub grouping of the PI cohort into individuals who were CFA+MF- or CFA+MF+ in 2009 did not reveal any differences. Nevertheless, another limitation of this study was the lacking diagnosis of soil transmitted helminths (STHs; ethical clearance did not cover this element) which were shown to induce Breg subsets in previous studies [12–14, 43–47]. However, our previous publication on immune profiling in the same region in Ghana showed low STH infection rates (6.3%) and ruled out co-infections as potential confounders [5]. Thus, we consider that the low prevalence of STH in the study region and the distribution of STH throughout all patient groups argues against STH being a bias for the findings in this study. In addition, a study about B cell subsets and their immune responses in *Schistosoma haematobium*-infected individuals in Gabon indicated that parasitic co-infections were also not an important confounder [67] Therefore, we suggest that the differences in Treg and Breg frequencies are not due to STHs and can be rather explained by the *W*. *bancrofti* infection, especially since Breg and Treg frequencies showed a positive correlation with MF counts. In addition, we performed a stepwise multiple logistic regression analysis and revealed only gender as confounder confirming previous results showing that females are more resistant to infection compared to men [68] and are outperforming their male counterparts in terms of MDA intake and thus compliance [69]. Indeed, rounds of MDA were not revealed as confounder showing that clearance of infection rather than rounds of treatment *per se* influence infection and thus frequencies of regulatory cell subsets.

In conclusion, this study presents initial evidence that IL-10-producing immature and B10 regulatory B cells were induced during an ongoing *W*. *bancrofti* infection in man, especially in patently infected individuals. These data contribute to the growing body of evidence about the complex nature of filarial-induced regulatory mechanisms in the host and indicate an important role of IL-10. In addition, MDA diminished the frequencies of regulatory immune cells in peripheral blood, suggesting that only active *W*. *bancrofti*-infections induce regulatory B cells in the periphery to shape host immunity. Further studies have to elucidate if re-infections of cured individuals (PI group) lead to enhanced induction of IL-10-producing immature and B10 regulatory B cells and the possible role of memory B cell activation. The capacity of filariae to modulate the host’s immune response is well reported but little is known about the long lasting impact of infection following cure. This study provided a unique opportunity to follow-up on a cohort after several years of MDA and moreover, compares regulatory cell profiles of those individuals with those presenting ongoing infections. The findings also revealed that distinct subsets of Breg populations were elevated during infection and moreover, that these populations had returned to base-line levels in the PI group. These data therefore provide initial evidence that certain filarial-specific cell populations are transient and decline following cure. Whether these are retained within the memory pool requires further investigation but it does underline that besides Treg populations, subsets of regulatory B cells play a crucial role within the complex host-filarial regulatory mechanisms and pathways.

## Supporting information

S1 Checklist STROBE checklist. (PDF)

S1 Fig Gating strategy for CD19^+^CD24^high^CD5^+^CD1d^high^ regulatory B cell populations. Peripheral blood cells were stained with fluorophore-conjugated anti-human CD1d, CD5, CD19 and CD24 monoclonal antibodies and frequencies of CD19^+^CD24^high^ and CD19^+^CD24^high^CD5^+^CD1d^high^ regulatory B cell populations were analysed according to the presented gating strategy.
(TIF)

S2 Fig Equal lymphocyte frequencies between the different groups. Using flow cytometry, peripheral whole blood cells from endemic normals (EN; n = 54), latent (CFA+MF-; n = 41) and patent (CFA+MF+; n = 13) *Wuchereria bancrofti*-infected, lymphedema (LE, n = 50) and previously infected individuals (PI; n = 65) were analyzed for frequencies (%) of lymphocytes. Graphs show box whiskers with median, interquartile ranges and outliers.
(TIF)

S3 Fig Gating strategy for CD19^+^CD5^+^CD1d^high^IL-10^+^ regulatory B cell populations. Peripheral blood cells were stained with fluorophore-conjugated anti-human CD1d, CD5, CD19 and IL-10 monoclonal antibodies and frequencies of CD19^+^CD5^+^CD1d^high^ and CD19^+^CD5^+^CD1d^high^IL-10^+^ regulatory B cell populations were analysed according to the presented gating strategy.
(TIF)

S4 Fig Gating strategy for CD19^+^CD24^high^CD38^high^IL-10^+^ regulatory B cell populations. Peripheral blood cells were stained with fluorophore-conjugated anti-human CD19d, CD24, CD38 and IL-10 monoclonal antibodies and frequencies of CD19^+^CD24^high^CD38^high^ and CD19^+^CD24^high^CD38^high^IL-10^+^ regulatory B cell populations were analysed according to the presented gating strategy.
(TIF)

S5 Fig Gating strategy for CD4^+^CD127^-^FOXP3^+^Neuropilin-1/HELIOS^+^ regulatory T cell populations. Peripheral blood cells were stained with fluorophore-conjugated anti-human CD4, CD127, FOXP3, HELIOS and Neuropilin-1 monoclonal antibodies and frequencies of CD4^+^CD127^-^FOXP3^+^Neuropilin-1^+^ and CD4^+^CD127^-^FOXP3^+^HELIOS^+^ regulatory T cell populations were analysed according to the presented gating strategy.
(TIF)

S1 Table Overall data set of the study. (XLSX)
