# Supplementary figures and images for: Wuchereria bancrofti-infected individuals harbor distinct IL-10-producing regulatory B and T cell subsets which are affected by anti-filarial treatment
Source: PLoS Negl Trop Dis. 2019 May 23;13(5):e0007436. doi: 10.1371/journal.pntd.0007436 (PMC6550419; doi:10.1371/journal.pntd.0007436)

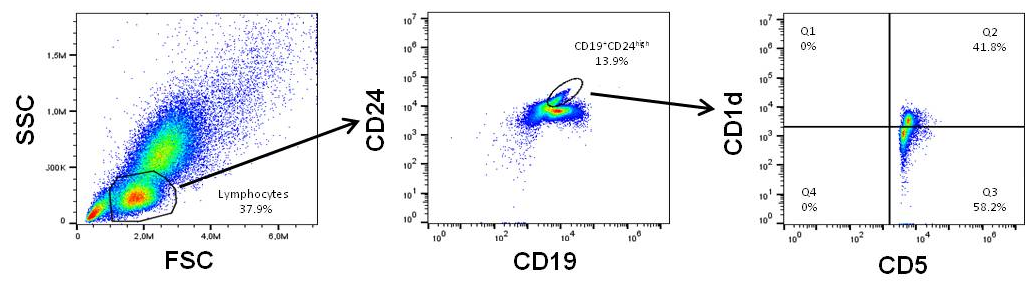

Supplement: S1 Fig — Peripheral blood cells were stained with fluorophore-conjugated anti-human CD1d, CD5, CD19 and CD24 monoclonal antibodies and frequencies of CD19+CD24high and CD19+CD24highCD5+CD1dhigh regulatory B cell populations were analysed according to the presented gating strategy. (TIF) [file pntd.0007436.s002.tif]

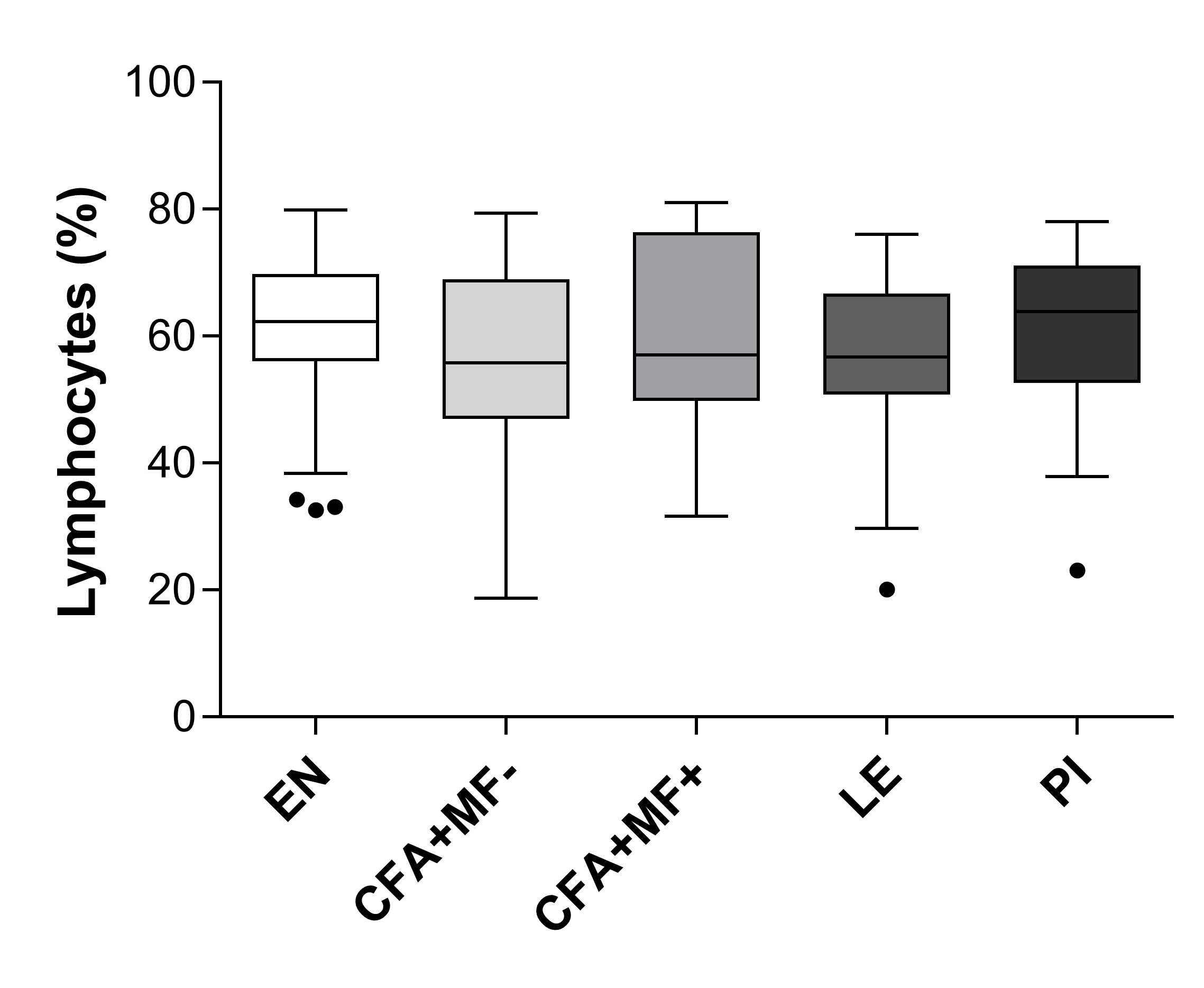

Supplement: S2 Fig — Using flow cytometry, peripheral whole blood cells from endemic normals (EN; n = 54), latent (CFA+MF-; n = 41) and patent (CFA+MF+; n = 13) Wuchereria bancrofti-infected, lymphedema (LE, n = 50) and previously infected individuals (PI; n = 65) were analyzed for frequencies (%) of lymphocytes. Graphs show box whiskers with median, interquartile ranges and outliers. (TIF) [file pntd.0007436.s003.tif]

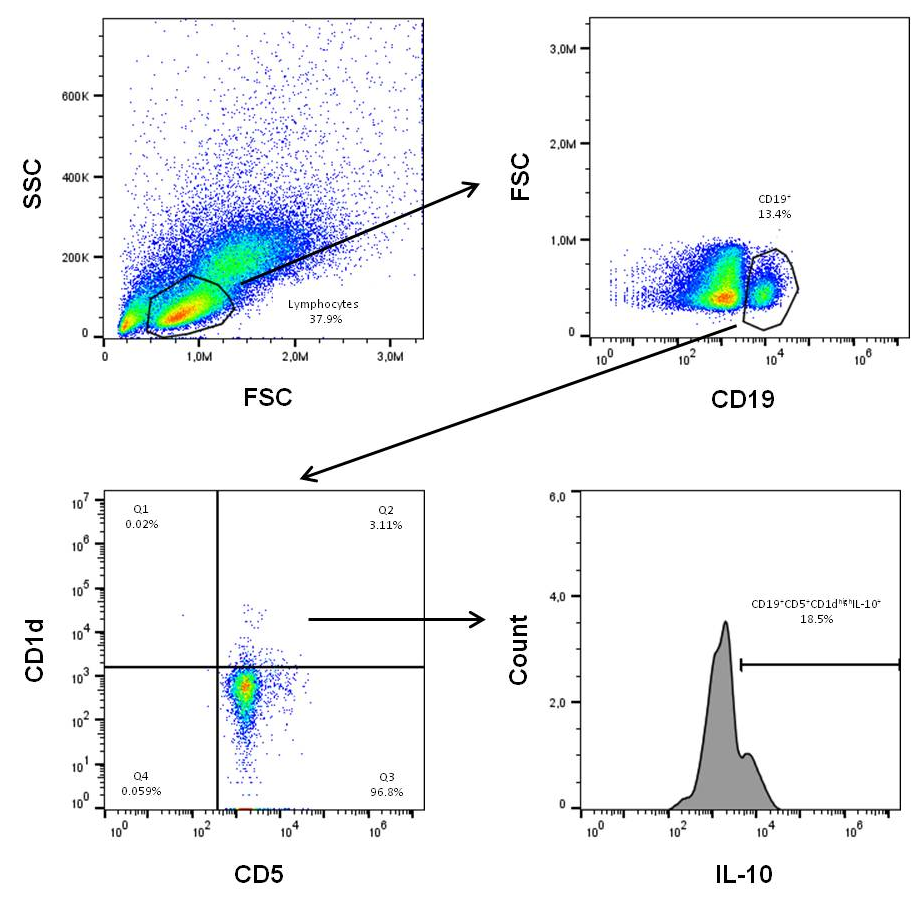

Supplement: S3 Fig — Peripheral blood cells were stained with fluorophore-conjugated anti-human CD1d, CD5, CD19 and IL-10 monoclonal antibodies and frequencies of CD19+CD5+CD1dhigh and CD19+CD5+CD1dhighIL-10+ regulatory B cell populations were analysed according to the presented gating strategy. (TIF) [file pntd.0007436.s004.tif]

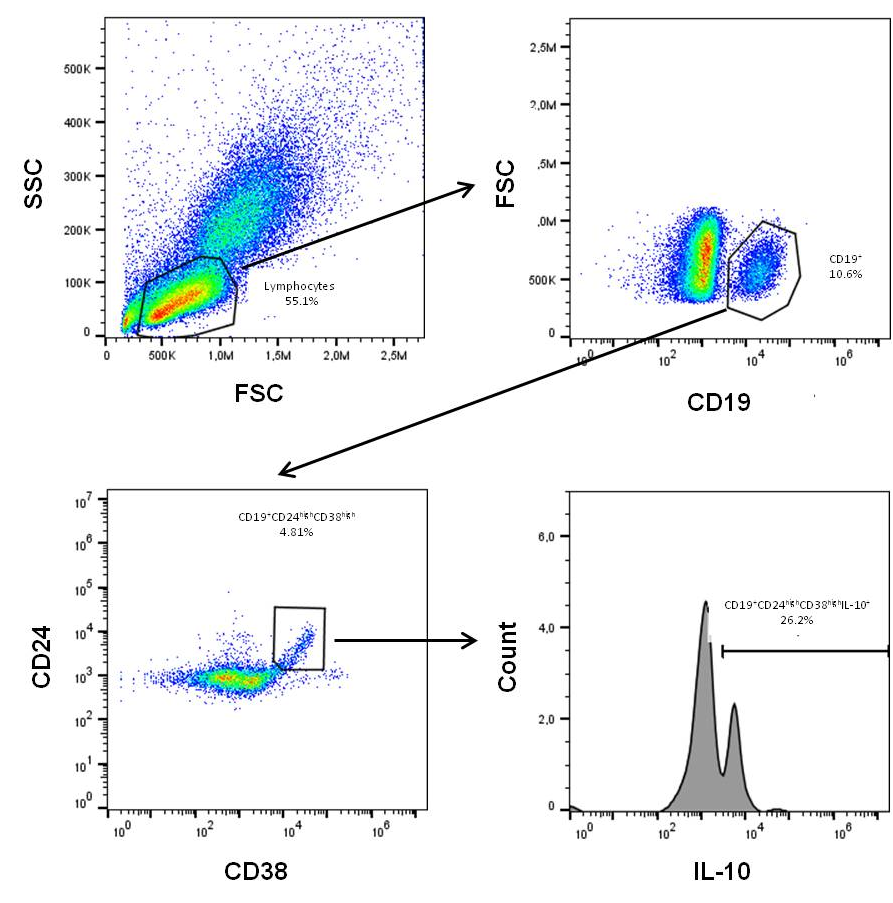

Supplement: S4 Fig — Peripheral blood cells were stained with fluorophore-conjugated anti-human CD19d, CD24, CD38 and IL-10 monoclonal antibodies and frequencies of CD19+CD24highCD38high and CD19+CD24highCD38highIL-10+ regulatory B cell populations were analysed according to the presented gating strategy. (TIF) [file pntd.0007436.s005.tif]

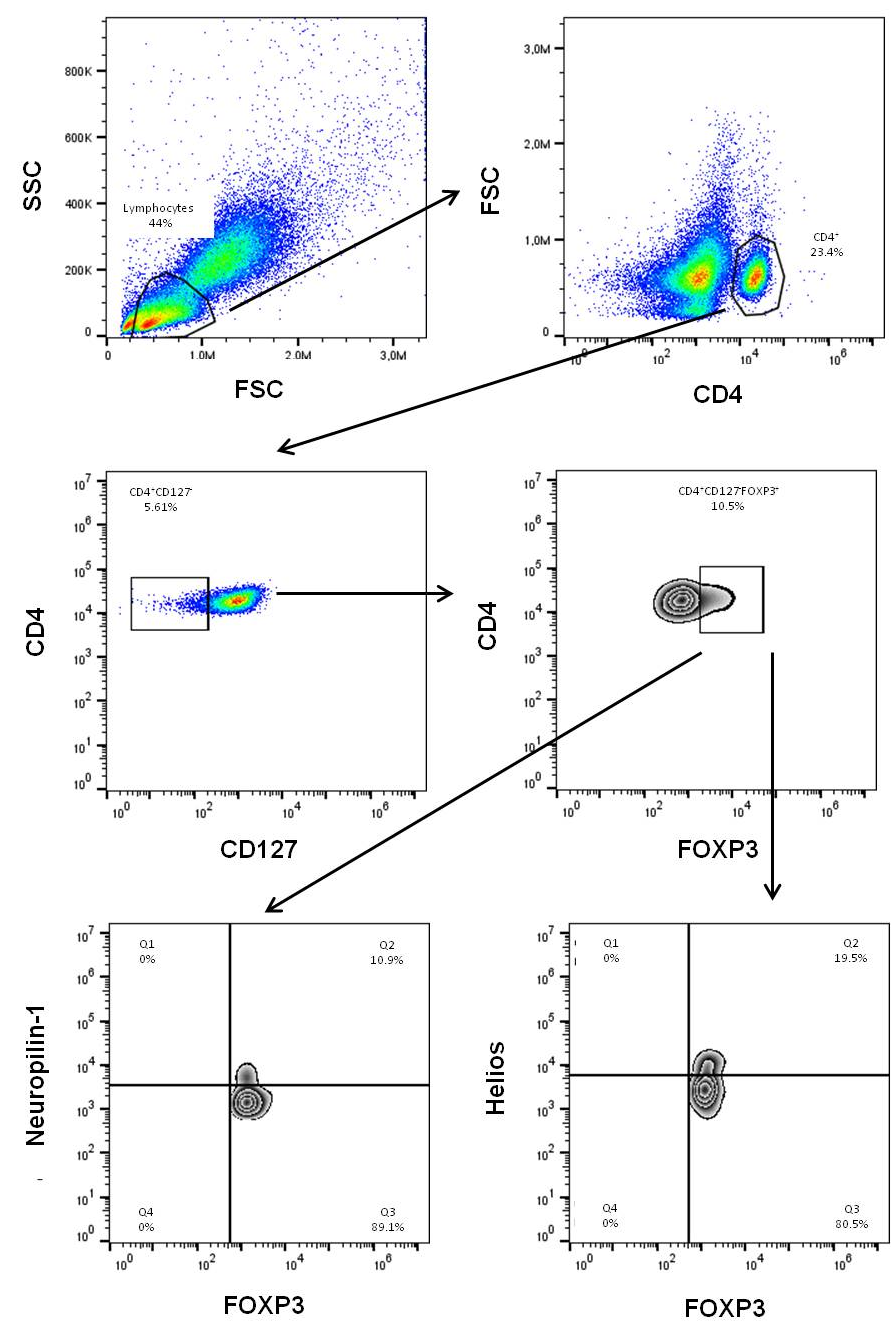

Supplement: S5 Fig — Peripheral blood cells were stained with fluorophore-conjugated anti-human CD4, CD127, FOXP3, HELIOS and Neuropilin-1 monoclonal antibodies and frequencies of CD4+CD127-FOXP3+Neuropilin-1+ and CD4+CD127-FOXP3+HELIOS+ regulatory T cell populations were analysed according to the presented gating strategy. (TIF) [file pntd.0007436.s006.tif]
